# The impact of juvenile idiopathic arthritis on psychosocial outcomes: a systematic review and meta-analysis

**DOI:** 10.1093/jpepsy/jsaf067

**Published:** 2025-08-05

**Authors:** Bethany Richmond, Louise Sharpe, Jack Boyse, Rachel E Menzies, Joanne Dudeney, Ruth Colagiuri, Jemma Todd

**Affiliations:** School of Psychology, The University of Sydney, Sydney, NSW, Australia; School of Psychology, The University of Sydney, Sydney, NSW, Australia; School of Psychology, The University of Sydney, Sydney, NSW, Australia; School of Psychology, The University of Sydney, Sydney, NSW, Australia; School of Psychological Sciences, Macquarie University, Sydney, NSW, Australia; Department of Rheumatology, Sydney Children’s Hospital Network, Sydney, NSW, Australia; Leeder Centre for Health Policy, Economics and Data, The University of Sydney, Sydney, NSW, Australia; Juvenile Arthritis Foundation Australia, Sydney, NSW, Australia; School of Psychology, The University of Sydney, Sydney, NSW, Australia

**Keywords:** juvenile idiopathic arthritis, psychosocial outcomes, psychological adjustment, health-related quality of life, mental health, chronic illness

## Abstract

**Objective:**

In addition to the physical challenges of juvenile idiopathic arthritis (JIA), youth with JIA also experience a range of psychosocial sequalae, which requires further attention. This meta-analysis aimed to compare the psychosocial outcomes of youth with JIA to healthy peers and other illness groups.

**Method:**

The protocol for this review was registered on PROSPERO (ID CRD42022348012). Seven electronic databases were searched from date of inception to February 20, 2024. Eligible studies reported on the health-related quality of life (HRQoL) or psychological outcomes of youth (<18 years) with JIA and included a comparison group. A modified Downs and Black Checklist was used to evaluate each study's quality. This research was supported by an Australian Government Research Training Program (RTP) Scholarship.

**Results:**

Fifty-six studies met inclusion criteria, representing a total of 335,708 participants. Compared to healthy controls, youth with JIA had more internalizing problems (*g *= 0.35, 95% confidence interval [CI] 0.13–0.57) and psychiatric diagnoses (*g *= 0.29, 95% CI 0.18–0.41), but did not differ on anxiety and depression symptoms. Relative to other illnesses, those with JIA had less anxiety (*g *= −0.26, 95% CI −0.42 to −0.10) and depressive symptoms (*g *= −0.51, 95% CI −0.73 to −0.30), but were similar in internalizing problems and psychiatric diagnoses. HRQoL was impaired relative to healthy peers overall (*g *= 0.74, 95% CI 0.46–1.02) and specifically in the physical domain (*g *= −0.89, 95% CI −1.18 to −0.60) and psychosocial domain (*g* = −0.53, 95% CI −0.68 to −0.37). Compared to other illnesses, youth with JIA had poorer physical (*g *= 0.79, 95% CI 0.03–1.55) and psychosocial HRQoL (*g *= 0.53, 95% CI 0.04–1.02). The quality of studies included were moderate but were limited by their poor external validity.

**Conclusions:**

Although youth with JIA had more internalizing problems and psychiatric diagnoses than healthy peers, they also demonstrated psychological resilience for anxiety and depression symptoms. However, they face large disparities in their HRQoL compared to both healthy youth and youth with other health conditions.

Juvenile idiopathic arthritis (JIA) affects more than 2 million youth worldwide (Dave et al., 2020), making it the most common chronic pediatric musculoskeletal condition (Ravelli & Martini, 2007). JIA is an umbrella term used to diagnose youth under 16 years with inflammatory arthritis that persists for 6 weeks or longer (Ravelli & Martini, 2007). There are six sub-types (Petty et al., 2004), and pain is a core symptom, with youth with JIA reporting pain on 70% of days (Bromberg et al., 2014; Schanberg et al., 2003; Timko et al., 1992). JIA is characterized by joint inflammation, which can result in abnormalities such as uneven limb lengths (Simon et al., 1981) and jaw asymmetry (Padeh et al., 2011; Twilt et al., 2004). The physical effects of JIA are not only to the musculoskeletal system, rather JIA can affect multiple bodily systems, such as vision loss due to uveitis (Foster et al., 2000; Thorne et al., 2010).

When youth with JIA experience pain, they withdraw more from social activities (Schanberg et al., 2003), attend school less consistently than other youth (Bouaddi et al., 2013), and report feeling stigmatized by peers (Tong et al., 2012). Studies confirm that youth with JIA experience greater social impairment, adjustment difficulties and internalizing problems than youth without JIA (Bomba et al., 2013; Memari et al., 2016; Mullick et al., 2005). A meta-analysis, more than two decades ago, found a higher risk of adjustment and internalizing problems among those with JIA relative to their healthy peers, but not more externalizing problems (LeBovidge et al., 2003). However, anxiety and depressive symptoms were not assessed separately in the LeBovidge et al. (2003) meta-analysis. Rather, in this meta-analysis, data were collected on broad measures of internalizing problems, primarily using the Child Behaviour Checklist (CBCL; Achenbach & Rescorla, 2001). Internalizing problems is an umbrella term including problems that are associated with distressing emotional experiences. The internalizing problems scale of the CBCL comprise items assessing anxiety and depression symptoms as well as somatic complaints. This makes it difficult to ascertain whether the differences in internalizing problems are due to increased anxiety and depression symptoms or somatic complaints. LeBovidge and colleagues recognized that the inclusion of somatic complaints may have inflated internalizing problems, since these are common among youth with health conditions due to the overlap with symptoms of their illness. As such, assessing anxiety and depression symptoms separately from internalizing problems will provide greater clarity on which particular symptoms underpin this disparity in youth with JIA.

Moreover, management of JIA has improved since that review, meaning a more up-to-date meta-analysis is needed. A systematic review (Fair et al., 2019) of 28 studies found that youth with JIA reported similar rates of anxiety and depression compared to youth with other illnesses, but increased rates in relation to youth without illness. This study did not quantify relationships through meta-analysis and included studies of adults with juvenile-onset arthritis. Neither of these reviews addressed health-related quality of life (HRQoL).

HRQoL provides an indication of the general health of an individual, encompassing their physical, psychological, and social functioning (Eiser & Morse, 2001; Karimi & Brazier, 2016). An assessment of HRQoL allows for the quantification of the burden of disease, pain, and disability. In youth with JIA, poorer HRQoL has been found to be associated with higher levels of pain, disability, and disease activity, less social support, worse school attendance, and more difficulties with treatment adherence (Haverman et al., 2012; Seid et al., 2014; Shaw et al., 2006). Understanding the impacts of JIA on HRQoL is crucial to estimate the overall impact of JIA on youth.

Not all studies have found impairments in HRQoL among youth with JIA (Grootenhuis et al., 2007; Listing et al., 2018). For example, Listing and colleagues (2018) initially found those with a recent JIA diagnosis had poorer HRQoL relative to youth without JIA. However, the HRQoL of 76% of youth with JIA became equivalent to youth without JIA within three years. Improved HRQoL may be due to the commencement of biologic therapies, which are associated with HRQoL (Anink et al., 2015; Kwon et al., 2015; Lovell et al., 2015). No meta-analysis has synthesized the HRQoL impacts of youth with JIA.

It is evident that youth with JIA face significant challenges, but the last review to meta-analysis psychosocial outcomes was conducted 20 years ago (LeBovidge et al., 2003). There is a crucial need to update our understanding of how JIA affects the mental health outcomes of young people. Furthermore, no review has quantified the impacts of JIA on HRQoL. The present review aims to address these gaps.

## Methods

### Search strategy and selection criteria

The protocol for this review was registered on PROSPERO (ID CRD42022348012, available at https://www.crd.york.ac.uk/PROSPERO/view/CRD42022348012). No major amendments were made to the protocol from registration. As outlined in the protocol, comparisons with control groups were planned. Due to the limited data across some outcomes, the meta-analysis evolved to focus more specifically on comparisons between JIA and both healthy and illness control groups. This meta-analysis was conducted in accordance with PRISMA guidelines (Page et al., 2021). The completed PRISMA checklist is available as Supplemental file 1. We searched seven databases as follows: MEDLINE/PubMed, EMBASE, PsycINFO, CENTRAL, Scopus, Web of Science, and CINAHL. Results were filtered to include only peer-reviewed studies with human populations, published in the English language. There was no restriction on the year published. The following search strategy was employed: [“juvenile arthritis” or “juvenile idiopathic arthritis” or “juvenile chronic arthritis” or “juvenile rheumatoid arthritis” or “juvenile rheumatic disease”], and [“psych*” or “psychosocial”, or “anx*” or “depress*” or “pain” or “quality of life”] (see Supplementary Table 1 for search strategy).

Studies were eligible for inclusion, if (i) participants were under 18 years, with a diagnosis of JIA, (ii) the study was original research, (iii) the impact of JIA on psychological functioning or HRQoL was examined, (iv) the outcomes of a control group or normative data, of either healthy peers or peers with another illness were reported, and (v) effect sizes for the comparisons between JIA and control groups could be calculated. Studies including participants over 18 years were included only if data were reported separately for those under 18. Titles and abstracts were independently screened by two authors (B. Richmond, J. Boyse). The full text of eligible studies was retrieved and independently reviewed by the same authors. Disagreements were resolved through discussion and the inclusion of a third reviewer (L. Sharpe).

### Data extraction and analysis

Data were extracted by two authors and included: Study details, participant characteristics (age, sex, sample size, disease type, disease duration), outcomes assessed, and measures used. For each outcome, mean and standard deviation were extracted for each group. However, for psychiatric diagnoses, the number of participants who either met criteria for any mental health disorder using a clinical interview or who had a mental health diagnosis on their medical records was used. Missing data were requested from the corresponding author. If authors did not respond or provide the requested data after two reminders, these data could not be included in analyses. Where studies utilized the same sample, the study with the largest number of participants was included. When studies provided both proxy and self-report for measures, self-report data were used.

Data were categorized under ‘internalizing problems’ outcome, where a broad measure of symptoms of emotional disturbance was used (e.g., the Child Behaviour Checklist [CBCL]; Achenbach & Rescorla, 2001), or where only the total score on a depression and anxiety symptom measure is provided (e.g., the Revised Children’s Anxiety and Depression Scale; Chorpita et al., 2000). When the depression and anxiety subscales were reported separately, or where a depression (e.g., Children’s Depression Inventory; Kovacs, 1985) or anxiety specific (e.g., State Trait Anxiety Inventory; Spielberger et al., 1971) measure was used, these were categorized under the anxiety symptoms and depressive symptoms outcomes. Another outcome measure of interest was externalizing problems, which were assessed using the CBCL or the Youth Self Report and encompasses the aggressive and rule-breaking behaviors subscales (Achenbach & Rescorla, 2001). Because self-report measures of anxiety and depressive symptoms do not indicate whether youth meet diagnostic criteria, we separately analyzed data from studies where semi-structured interviews or medical records were used to classify psychiatric disorders. Hence, data on psychiatric diagnoses conveys information about the number of participants who met criteria for any mental health disorder which is helpful to determine whether increased symptoms are of clinical significance.

The HRQoL outcomes included studies that assessed HRQoL using validated measures. When reported, a total HRQoL score was extracted and analyzed. In studies that provided a psychosocial summary score, as commonly calculated when using the Pediatric Quality of Life Inventory (PedsQL; Varni et al., 2001), this was extracted and used as the psychosocial HRQoL outcome. A physical HRQoL outcome was also extracted from studies that reported a separate physical HRQoL domain score. Where studies reported a total HRQoL score, alongside a psychosocial and physical summary score, we analyzed all outcomes separately. While this means that some analyses are not entirely independent, this approach allowed us to better characterize the nature of differences between groups across these domains.

For studies that reported on subgroups, such as type of JIA or age of the child, combined data were used where available. In instances where studies included multiple control groups, the JIA sample size was divided by the number of groups to ensure that additional weight was not given to these studies. Baseline data were extracted from longitudinal studies, and follow-up data were only used when baseline data were not provided.

All analyses were completed using Comprehensive Meta-Analysis (CMA; Borenstein et al., 2022). Where there were at least three independent studies available, means and standard deviations were used to calculate Hedge’s *g* (Hedges, 1981) and 95% confidence intervals (CI) using the random effects model. Positive effect sizes indicate that the mean score for the JIA group is higher than the comparator group. A negative effect size indicates that the JIA group scored lower on average on the outcome relative to the comparator group. Heterogeneity was considered significant if *I*^2^ was >50% or if *p* < .05 for Cochrane’s Q. If heterogeneity was significant, and provided there were sufficient studies (i.e., 3 or more), meta-regressions were conducted to explore possible variables contributing to heterogeneity, such as JIA sample age, proportion of females, duration of disease, measure used and illness type. The risk of publication bias was evaluated using Egger’s weighted regression (Egger et al., 1997) and Begg–Mazmudar rank correlation (Begg & Mazumdar, 1994). If either of these tests were significant, Duval and Tweedie Trim and Fill analysis (Duval & Tweedie, 2000) would be used to adjust for publication bias. Study characteristics and the main meta-analytic findings were summarized in tables created in Microsoft Word. Forest plots were created in CMA which included the effect size and 95% CI for overall analysis and individual studies. A table summarizing each studies risk of bias was also created using RobVis (McGuinness & Higgins, 2021). Data are available on request.

Treatment for JIA has improved drastically since 1999, with the approval of the first biologic medication, Etanercept (Lovell et al., 2000). By including studies prior to this date, our results may overestimate the psychosocial impact of JIA. To account for this, we conducted a post hoc sensitivity analysis, removing studies published before or during 2000. In addition to this, we ran a post hoc sensitivity analysis, excluding studies which were published before or during 2003, to look only at studies published in the time since the last meta-analysis (LeBovidge et al., 2003).

The quality of studies was evaluated independently by two reviewers (BR, JB) using a modified version of the Downs and Black Quality Index Scale (QIS) (Downs & Black, 1998). This version was adapted by Ferro & Speechley (2009) to better evaluate the methodological quality of observational studies. The modified QIS has 15 items across four domains: reporting, external validity, internal validity, and power. For each item, studies were awarded a score of 1 if they met the requirement, or a 0 which indicated insufficient reporting or unable to determine. Scores were added together to provide an overall QIS score, with higher scores suggesting better quality research. Disagreement was resolved by consensus. The confidence in each analysis was also evaluated using the Grading of Recommendation, Assessment, Development and Evaluation (GRADE) approach and guidelines described by Cochrane (Schünemann et al., 2023).

## Results

**Figure 1.**
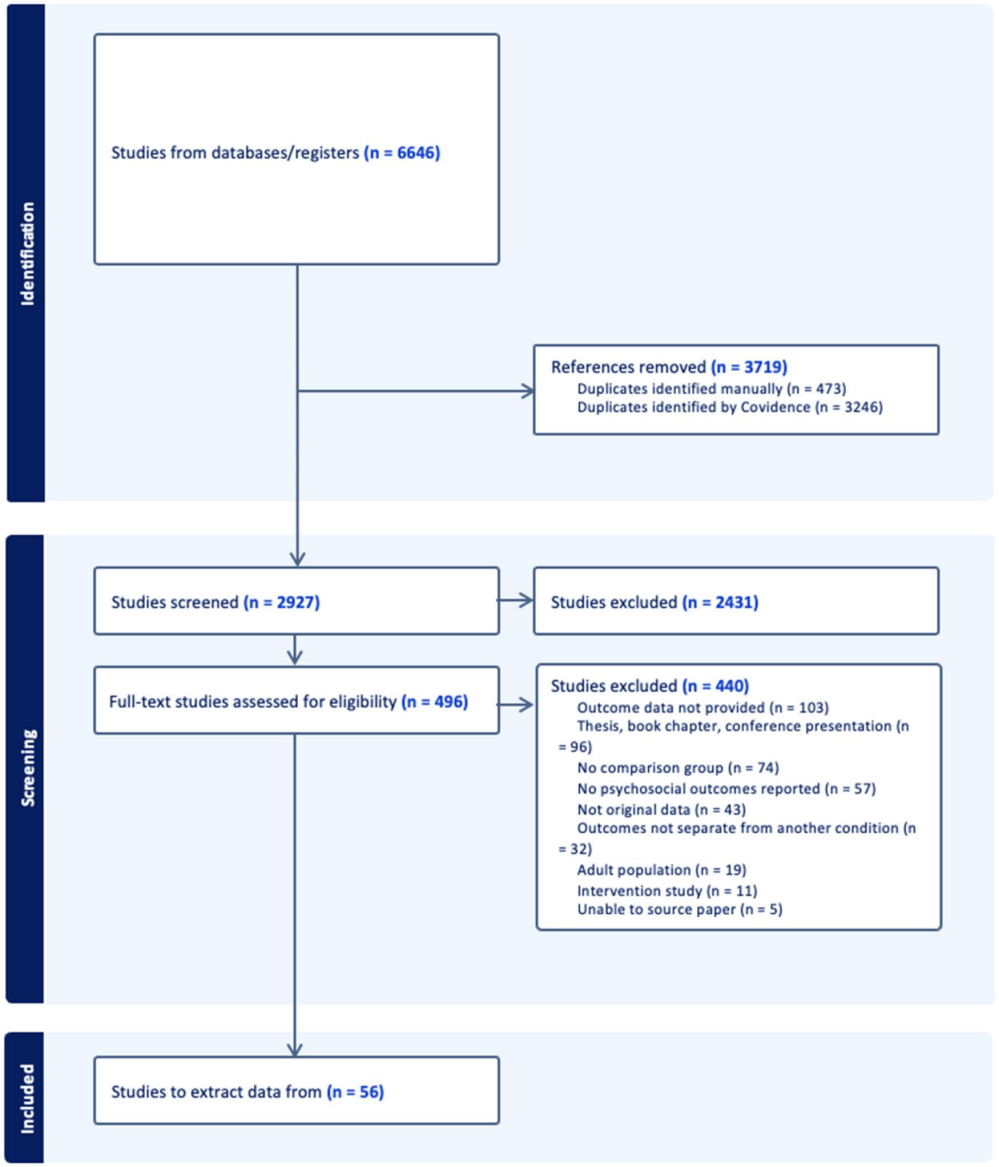
PRISMA diagram. Created by the authors in accordance with PRISMA 2020 guidelines (Page et al., 2021).

Following our searches of the databases, a total of 6,646 studies were identified (see Figure 1). Of these, 3,719 were duplicates. Following title and abstract screening, 496 full-text studies were obtained. Of these, 56 studies met the inclusion criteria and were included in our review. Common reasons for exclusion included: no data on psychosocial outcomes (*n* = 160), publication type (i.e., thesis, conference abstract) (*n* = 96), no comparison group (*n* = 74), no original data reported (*n* = 43), outcomes were not separate from another condition (*n* = 32) or an adult only sample was used (*n* = 19).

The number of participants ranged from 40 to 201,686, with a median of 117.5 (IQR = 302.5) (Table 1). The mean age of participants with JIA was 11.51 (*SD *= 2.51), with average disease duration of 4.87 years (*SD *= 1.75). Youth with JIA were more frequently female (*M *= 64.62%, *SD *= 12.28). The mean age of the control groups was comparable to youth with JIA (*M *= 11.97, *SD *= 2.48); however, the percentage of female youth was lower (*M = *57.78%, *SD *= 15.31). Thirty-eight studies included a healthy control group, and 31 studies included at least one illness group. Fourteen studies featured both a healthy control and an illness group. The illness most represented in control groups were diabetes (*k *= 6), chronic fatigue syndrome (CFS) (*k *= 5), and sickle cell disease (SCD) (*k *= 4).

**Table 1.** Summary of study characteristics.

Study	Design	Country	Ethnicity/Race	SES	Outcome measures	Subgroup	JIA N	Mean age	Female	Mean disease duration (years)	Control group	Control group N	QIS
Aasland et al. (1997)	Longitudinal	Norway	n/a	JIA mother’s education: median 10.8 years, range (7–19), father’s education: median 11, range (7–19) Controls Mother’s education: median 12 years, range (8–20), father’s education: 10, range (8–19)	Psychiatric Diagnoses—CAS	n/a	52		56%	n/a	Idiopathic musculoskeletal pain	23	7
Aasland & Diseth (1999)	Cross-sectional	Norway	n/a	Mothers education: <10 years = 27.9%; 10–12 years = 44.2%; >12 years = 27.9%. father’s education: <10 years = 20.9%; 10–12years = 39.5%; >12 years = 34.9%; unknown = 4.7%	Externalizing—YSR Internalizing—YSR Psychiatric diagnoses—CAS	n/a	23	14.4	52%	n/a	Anorectal abnormalities	20	10
Abdelaleem et al. (2021)	Cross-sectional	Egypt	n/a	n/a	Functional disability—CHAQ HRQoL– PedsQL Pain—VAS	n/a	25	11.48	72%	n/a	Healthy	25	7
Bomba et al. (2013)	Cross-sectional	Italy	n/a	n/a	Anxiety—SAFA-A Depression—CDI HRQoL—PedsQL	n/a	39	11.43	69.2%	3.15	Healthy	80	8
Brace et al. (2000)	Cross-sectional	United States	n/a	JIA SES = 43.1 (*SD* = 15.7) Healthy Control SES = 44.2 (*SD* = 15.4) CFS SES = 43.6 (*SD* = 18.6)	Depression—CDRS Internalizing—YSF	n/a	16	14.25	75%	n/a	Healthy	14	11
											CFS	10	
Butbul Aviel et al. (2011)	Cross-sectional	Israel	n/a	n/a	HRQoL—PedsQL Pain—VAS	Oligoarthritis	31	12.5	71%	n/a	Juvenile dermatomyositis	33	13
						Polyarticular arthritis	33	12.9	76%	n/a			
						Systemic arthritis	28	12.7	50%	n/a			
Butler et al. (2018)	Cross-sectional	Canada	94% Caucasian	Income ≥$C90,000 = 58%	Psychiatric diagnoses—MINI-KID	n/a	11	11.3	n/a	n/a	Mixed	39	13
Castaneda et al. (2013)	Cross-sectional	Finland	n/a	SES = Class I = 27.3%; Class II = 27.3%; Class III = 43.2%; Class IV = 2.3%	Depression—BDI	n/a	23	15.5	61%	n/a	IBD	34	13
Conte et al. (2003)	Cross-sectional	United States	n/a	n/a	Anxiety—STAI-T Depression—CDI Externalizing—CBCL Internalizing—YSR	n/a	16	12.6	63%	n/a	FMS	16	9
											Healthy	16	
Delcoigne et al. (2023)	Cross-sectional	Sweden	Country of birth: Sweden = 94.5%; Other = 5.5%	Parent’s education: primary school = 3.4%; secondary school = 41.2%; higher education = 55.4%	Psychiatric diagnoses—records	n/a	3717		62.4%	n/a	Healthy	17,715	11
Feldmann et al. (2005)	Cross-sectional	Germany	n/a	SES: upper middle = 22.6%; middle = 61.3%; lower = 16.1%	Externalizing—CBCL Internalizing—CBCL	n/a	31	12.5	48%	6.2	Healthy	31	7
Filocamo et al. (2010)	Cross-sectional	Italy	n/a	n/a	HRQoL—PRQL	n/a	472	4.2	77%	5	Healthy	800	12
Fischer et al. (2019)	Cross-sectional	France	n/a	High SES = 26.7%; medium SES = 70.4%; low SES = 2.8%	HRQoL—KidsCAT	n/a	49	12.29	71%	4.24	Asthma	58	10
											Diabetes	202	
Fontecha et al. (2011)	Cross-sectional	Spain	n/a	Family affluence score: low = 16.3%; medium = 43.3%; high = 40.4%	HRQoL—KIDSCREEN	n/a	27	13.1	80.8%	n/a	Healthy	152	10
											Low back pain patients	76	
											Low back pain school	152	
Frank et al. (1998)	Cross-sectional	United States	Caucasian = 97.4%; Other = 2.6%	SES 37.37 (*SD* = 15.49)	Internalizing—CAS	n/a	107	8.26	65%	2.19	Diabetes	114	10
											Healthy	88	
Fuchs et al. (2013)	Cross-sectional	The Netherlands	Caucasian = 93.1%; African = 3.9%; Latin American = 2 1.7%; Asian = 1%	n/a	HRQoL—CHQ Pain—CHQ	n/a	37	16.3	84%	n/a	Healthy	23	10
											CFS	37	
Gartstein et al. (1999)	Cross-sectional	United States	White = 70%; African American = 30%	Family SES for chronic illness group = 35.15; SES healthy controls = 35.35	Externalizing—CBCL Internalizing—CBCL	n/a	35		68.6%	n/a	Healthy	35	11
											SCD	49	
											Hemophilia	20	
											Cancer	64	
Gray et al. (2001)	Cross-sectional	United States	n/a	Mean SES = 1.9 (SD = 0.8)	Externalizing—CBCL Internalizing—CBCL	n/a	15	12.7	93%	n/a	CFS	15	8
Graziano et al. (2016)	Cross-sectional	Italy	n/a	Mother’s educational status: High school or lower = 80%; University or higher = 20%	externalizing—CBCL Internalizing—CBCL	1-5 years	6	6.9	50%	n/a	Cancer	7	10
											Healthy	7	
						6-18 years	9	6.9	50%	n/a	Cancer	8	
											Healthy	8	
Guerriero et al. (2022)	Cross-sectional	Italy	n/a	n/a	Externalizing—CBCL HRQoL—PedsQL Internalizing—CBCL	n/a	30	9.7	56.7%	n/a	Healthy	30	10
Haines et al. (2019)	Cross-sectional	United Kingdom	n/a	n/a	Anxiety—RCADS Depression—RCADS	n/a	42	13.86	81%	n/a	CFS	49	11
											Type 1 diabetes	52	
Haverman et al. (2012)	Cross-sectional	The Netherlands	n/a	n/a	HRQoL—PedsQL	Young Children	14	n/a	n/a	n/a	Healthy	61	12
											Chronic illness	11	
						Children	63	n/a	n/a	n/a	Healthy	192	
											Chronic illness	26	
						Adolescents	75	n/a	n/a	n/a	Healthy	148	
											Chronic illness	25	
Huygen et al. (2000)	Cross-sectional	The Netherlands	n/a	Lower class SES = 31.3%	Anxiety—SASK Depression—DSRI externalizing—CBCL Internalizing—CBCL	Children	23	n/a	68%	5.63	Healthy	25	9
						Adolescents	25	n/a	68%	6.78	Healthy	27	
Janicke et al. (2008)	Cross-sectional	The United States	White = 30%; African American = 29.4%; Hispanic = 27.2%; Native American = 0.1%; Asian American = 0.4%; Other = 12%	n/a	Psychiatric diagnoses—records	n/a	584	9.9	62%	n/a	Metabolic syndrome	3,030	10
											Type 2 diabetes	3,066	
											Dyslipidemia	4,057	
											Asthma	46,157	
											Cystic fibrosis	393	
											SCD	2,496	
Kayan Ocakoglu 2018	Cross-sectional	Turkey	n/a	n/a	Anxiety—SCARED Depression—CDI Externalizing—CBCL Internalizing—CBCL Psychiatric diagnoses—KSADS	n/a	34	14.25	28.10%	n/a	Healthy	30	7
											Immunodeficiency disorder	44	
Kuburovic et al. (2014)	Cross-sectional	Serbia	n/a	Mothers employed = 71.3%, unemployed = 28.7%; fathers employed = 74.4%; unemployed = 25.6%	Anxiety—SCARED Depression—MFQ HRQoL—PedsQL	n/a	50	11.4	52%	n/a	Healthy	77	11
											Immunodeficiency disorder	19	
Kwon et al. (2015)	Cross-sectional	Korea	n/a	n/a	Functional disability—CHAQ HRQoL—PedsQL	n/a	26	n/a	42.3%	n/a	Healthy	25	8
Kyllönen et al. (2021)	Cohort	Finland	n/a	n/a	Psychiatric diagnoses—records	n/a	4180	8.3	62%	n/a	Healthy	12,512	9
Listing et al. (2018)	Longitudinal	Germany	Country of origin, Germany = 76.9%	Low SES = 25.3%; Moderate SES = 50.1%; High SES = 24.5%	HRQoL—PedsQL Pain—PedsQL	n/a	953	8.4	59.1%	n/a	Healthy	491	10
Long et al. (2008)	Cross-sectional	United States	Caucasian = 57.7%; African American = 40.4%; Other = 1.9%	Family income <$10,000 = 13.4%; $10,000–29,000 = 23.7%; $30,000–49,000 = 19.6%; $50,000–69,000 = 18.6%; >$70,000 = 24.7%	HRQoL—CHQ	n/a	30	10.22	56%	n/a	Headache	44	10
											SCD	26	
Lundberg & Eriksson (2017)	Cross-sectional	Sweden	n/a	n/a	HRQoL—DISABKIDS	n/a	53	n/a	71.7%	n/a	Mixed	1152	11
McDonald et al. (2022)	Cross-sectional	United States	White = 81.2% African American = 13.5%; Asian = 3.5%); Muli-racial = 2.6%); American Indian/Alaskan Native = 1.8%; Unknown/declined = 1.8%; Hispanic = 7.5%	n/a	Functional disability—CHAQ HRQoL—PedsQL Internalizing—RCADS	n/a	332	10.4	71%	n/a	Non-anterior uveitis	45	10
											Anterior uveitis	48	
Milatz et al. (2024)	Cohort	Germany	n/a	n/a	Psychiatric diagnoses—records	12–14 years	182	13	71%	n/a	Healthy	1820	10
						15–17 years	234	15.9	68%	n/a	Healthy	2340	
Mullick et al. (2005)	Cross-sectional	Bangladesh	n/a	Low income = 6.7%; Middle income = 80%; High income = 13.3%	Psychiatric diagnoses—ICD-10 clinical interview	n/a	40	13.25	40%	2.7	Healthy	40	10
Noll et al. (2000)	Cross-sectional	United States	n/a	Mean SES JIA: 47.40 (SD = 21.90) Mean SES Control: 46.71 (SD = 20.34)	Depression—CDI Internalizing—CBCL	n/a	74	11.07	54%	5.81	Healthy	74	13
Ojmyr-Joelsson et al. (2006)	Cross-sectional	Sweden	n/a	n/a	Externalizing—CBCL Internalizing—CBCL	n/a	26	10.6	83%	n/a	Healthy	31	6
											Imperforate anus	24	
Oliveira et al. (2007)	Cross-sectional	Multi-national	n/a	n/a	Functional disability—CHAQ HRQol—CHQ Pain—VAS	n/a	3324	10	68%	4.1	Healthy	3315	8
Oymak et al. (2015)	Cross-sectional	Turkey	n/a	Household income equal/more than living expenses = 27.4%; less than living expenses = 72.6%	Functional disability—FISH HRQoL—KINDL	n/a	19	n/a	n/a	n/a	Healthy	32	7
											Hemophilia	33	
Pedersen et al. (2024)	Cohort	Denmark	n/a	Family income: low = 32.2%; middle = 33.6%; high = 33.9%; missing = 0.4%	Psychiatric diagnoses—records	n/a	2086	13.4	62.7	n/a	Healthy	208,600	11
Peterson & Palermo (2004)	Cross-sectional	United States	White = 60%; African American = 29%; Hispanic = 2%; Other = 1%	Family income: <10,000 = 12%; 10,000–19,000 = 7%; 20,000–29,000 = 10%; 30,000–39,000 = 13%; 40,000–49,000 = 6%; 50,000–59,000 = 11%; 60,000–69,000 = 8%; >70,000 = 28%	Anxiety—RCADS Depression—RCADS Pain—FPS Functional disability—FDI	n/a	63	12.4	60%	n/a	Headache	98	11
											SCD	54	
Polat et al. (2024)	Cross-sectional	Turkey	n/a	n/a	HRQoL—PedsQL	n/a	50	11.8	66%	4	Healthy	50	10
Rangel et al. (2003)	Cross-sectional	United Kingdom	Caucasian = 86.2%; Black = 1.7%; Asian (southeast) = 5.2%; Mixed = 3.4%; Other = 3.4%	Social Class: 1 = 12.1%; II = 53.4%; III = 22.4%; IV = 8.6%	externalizing—CBCL Internalizing—KSADS Psychiatric diagnoses—CBCL	n/a	28	15.2	67%	9.8	CFS	25	10
Reid et al. (1997)	Cross-sectional	Canada	n/a	n/a	Anxiety—STAI-T Depression—CDI Functional Disability—FDI Pain—NRS	n/a	15	14.5	87%	5.8	Healthy	15	9
											FMS	15	
Ringold et al. (2009)	Cross-sectional	United States	n/a	n/a	HRQoL—PedsQL	n/a	55	8.4	83%	n/a	Healthy	5079	12
Scott et al. (2018)	Cross-sectional	South Africa	n/a	n/a	HRQoL—JAMAR	n/a	142	10.88	62%	n/a	Healthy	122	8
Selvaag et al. (2005)	Longitudinal	Norway	n/a	Parents with >12 years education = 31.5%; Parents employed = 79.3%	HRQoL—CHQ	n/a	173	9.3	60.3%	n/a	Healthy	116	12
Shaaban et al. (2006)	Cross-sectional	Egypt	n/a	n/a	Functional disability—CHAQ HRQoL—CHQ Pain—CHQ	n/a	52	n/a	59.62%	3.48	Healthy	61	8
Sikorová & Buzgova (2016)	Cross-sectional	Czech Republic	n/a	n/a	HRQoL—PedsQL	n/a	64	n/a	n/a	n/a	Asthma	75	8
											Diabetes	81	
											Epilepsy and eczema	68	
Tarakci et al. (2011)	Cross-sectional	Turkey	n/a	n/a	Anxiety—SCARED Depression—CDI Functional Disability—CHAQ	n/a	52	12.3	64%	5.39	Healthy	48	11
Tsipoura et al. (2018)	Cross-sectional	Greece	n/a	n/a	HRQoL—KINDL	n/a	50	11.4	70%	n/a	Healthy	50	10
Wagner et al. (2007)	Cross-sectional	United States	Caucasian = 46%; Native American = 26%; Hispanic = 10%; African American = 8%; Biracial = 8%; Asian = 2%	n/a	Depression—CDI	n/a	29	n/a	n/a	n/a	Rheumatic diseases	21	9
Wallander et al. (1988)	Cross-sectional	United States	n/a	Family income: State aid only = 9%; <10,000 = 14%; 10,000–20,000 = 28%; 20,000–30,000 = 17%; 30,000–40,000 = 10%; >40,000 = 21%	Externalizing—CBCL Internalizing—CBCL	n/a	24	10.3	46%	n/a	Diabetes	80	9
											Spina bifida	77	
											Hemophilia	40	
											Obesity	30	
											Cerebral Palsy	19	
Ward et al. (2017)	Cross-sectional	United States	White = 73.3%; Mixed = 14.7%; Asian American = 8.4%; African American = 1.4%; Hispanic/Latio = 4.2%	n/a	HRQoL—PedsQL	n/a	46	8.5	55.9%	3.15	Healthy	56	11
Ward et al. (2014)	Cross-sectional	United States	White = 78.1%; Asian = 10.5%; Other = 2.6%	n/a	Externalizing—CBCL Internalizing—CBCL	n/a	68	8.5	74%	3.6	Healthy	46	8
Weitzman et al. (2023)	Cross-sectional	United States	White = 75%; Asian = 4.5%; African American = 4.2%; more than one race = 4.5%; Other = 3.5%; Unknown = 8.3%	Parent education: less than college degree = 10.1%; college degree or higher = 55.7%; missing = 34.2%	Anxiety—PROMIS Depression—PROMIS	n/a	366	13.6	69.1%	5.52	Systemic lupus erythematosus	58	12
Zebracki et al. (2004)	Cross-sectional	United States	Non-minority = 94%; minority = 6%	n/a	HRQoL—CHQ Pain—CHQ	n/a	36	10.9	75%	n/a	Healthy	36	12
											Primary Immunodeficiency	36	

*Note. CFS* = chronic fatigue syndrome; *CG* = control group; *CHQ* = The Child Health Questionnaire; *FMS* = fibromyalgia syndrome; *HRQoL* = health-related quality of life; *IBD* = irritable bowel syndrome; *JAMAR* = Juvenile Arthritis Multidimensional Assessment Report; *JIA* = juvenile idiopathic arthritis; *N* = number of participants; *n/a* = not available/not applicable; *SES* = socioeconomic status; *PedsQL* = The Pediatric Quality of Life Inventory; *PRQL* = The Pediatric Rheumatology Quality of Life Scale; *QIS* = quality index score; *SCD* = sickle cell disease;

Compared to healthy controls, young people with JIA had significantly more internalizing problems (*g *= 0.35, 95% CI 0.13–0.57, *p *= .002) and higher rates of psychiatric diagnoses (*g *= 0.29, 95% CI 0.18–0.41, *p* < .001) (Table 2) (see Supplementary materials for forest plots). No difference in anxiety, depressive symptoms, or externalizing problems was identified between youth with and without JIA.

**Table 2.** Psychosocial outcomes in JIA compared to healthy controls.

Outcome	No. of studies	No. of effects	Effect size (95% CI)	*p*	*Q*	*I^2^*
Internalizing	12	14	0.35 (0.13 to 0.57)	.002	25.38	48.78
Psychiatric diagnoses	6	7	0.29 (0.18 to 0.41)	<.001	42.74	85.96
Anxiety	7	7	0.29 (−0.02 to 0.61)	.069	11.58	48.21
Depression	9	10	0.19 (−0.13 to 0.51)	.234	26.83	66.45
Externalizing	9	11	0.15 (−0.05 to 0.34)	.135	11.28	11.34
Total HRQoL	15	17	−0.74 (−1.02 to −0.46)	<.001	211.49	92.44
Physical HRQoL	19	21	−0.89 (−1.18 to −0.60)	<.001	571.22	96.50
Psychosocial HRQoL	11	13	−0.53 (−0.68 to −0.37)	<.001	81.79	85.33
Pain	8	8	1.25 (1.07 to 1.43)	<.001	27.03	74.10
Functional Ability	7	7	1.20 (0.95 to 1.45)	<.001	14.12	57.50

The JIA group had significantly poorer total HRQoL relative to their healthy peers (*g* = −0.74, 95% CI −1.02 to −0.46, *p* < .001) with a large discrepancy in the physical domain (*g *= −0.89, 95% CI −1.18 to −0.60, *p* < .001) and a moderate deficit in the psychosocial domain (*g* = −0.53, 95% CI −0.68 to −0.37, *p* < .001). Relative to healthy controls, youth with JIA had significantly more pain (*g *= 1.25, 95% CI 1.07 − 1.43, *p* < .0001) and greater disability (*g *= 1.2, 95% CI 0.95 − 1.45, *p *< .001).

Internalizing problems and rates of psychiatric diagnoses did not differ between youth with JIA and other illnesses (Table 3). However, those with JIA had significantly less depressive (*g *= −0.51, 95% CI −0.73 to −0.30, *p* < .001) and anxiety symptoms (*g* = −0.26, 95% CI −0.42 to −0.10, *p *= .001) and had fewer externalizing problems (*g *= −0.25, 95% CI −0.45 to −0.04, *p *= .020) compared to other illness groups.

**Table 3.** Psychosocial outcomes in JIA compared to illness controls.

Outcome	No. of Studies	No. of Effects	Effect size (95% CI)	*p*	*Q*	*I^2^*
Internalizing	12	20	−0.31 (−0.82 to 0.20)	.232	223.70	91.51
Psychiatric diagnoses	6	11	−0.13 (−0.29 to 0.03)	.114	22.91	56.35
Anxiety	7	9	−0.26 (−0.42 to −0.10)	.001	5.63	0
Depression	10	12	−0.51 (−0.73 to −0.30)	<.001	19.77	44.36
Externalizing	9	16	−0.25 (−0.45 to −0.04)	.020	17.02	11.85
Total HRQoL	7	14	−0.68 (−1.46 to 0.11)	.093	417.23	96.88
Physical HRQoL	10	16	−0.79 (−1.55 to −0.03)	.042	490.29	96.94
Psychosocial HRQoL	4	8	−0.53 (−1.02 to −0.04)	.035	51.52	86.41
Pain	5	8	−0.39 (−1.01 to 0.22)	.213	60.00	88.33
Functional ability	4	6	−0.009 (−0.47 to 0.45)	.971	30.84	83.79

Total HRQoL did not differ between youth with JIA and other illnesses. However, those with JIA had significantly poorer physical (*g *= −0.79, 95% CI −1.55 to −0.03, *p *= .042) and psychosocial HRQoL (*g *= −0.53, 95% CI −1.02 to −0.04, *p *= .035) than other illness groups. No difference in pain ratings nor functional abilities emerged between young people with JIA and other illnesses.

Moderation analyses revealed that studies with a higher average age of JIA participants were associated with a reduced disparity in psychiatric diagnoses (*B *= −0.14, 95% CI −0.23 to −0.04, *p *= .004), total (*B *= −1.51, 95% CI −2.82 to −0.19, *p *= .025), and physical HRQoL (*B *= −0.74, 95% CI −1.44 to −0.04, *p *= .039) (Table 4). There were insufficient studies to assess differences in JIA disease duration, measure used and illness type on most outcomes. However, a longer disease duration was associated with fewer depressive symptoms (*B *= −0.29, 95% CI −0.46 to −0.12, *p *= .001) but more internalizing problems (*B *= 0.13, 95% CI 0.003–0.25, *p *= .045) relative to healthy peers.

**Table 4.** Significant moderators.

Outcome	Control	Moderator	*B* (95% CI)	*p*	*R* ^2^
Psychiatric diagnoses	Illness	Age	−0.14 (−0.23 to −0.04)	.004	0.61
Total HRQoL	Illness	Age	−1.51 (−2.82 to −0.19)	.025	0.53
Physical HRQoL	Illness	Age	−0.74 (−1.44 to −0.04)	.039	0.26
Internalizing	Healthy	Disease duration	0.13 (0.003 to 0.25)	.045	1
Depression	Healthy	Disease duration	−0.29 (−0.46 to −0.12)	.001	1

There was no evidence of publication bias for all analyses according to both Egger’s regression (*p*s ≥ .06) and Begg–Mazumdar rank tests (*p*s ≥ .11). QIS scores ranged from 6 to 13, with a mean score of 9.77 (*SD *= 1.70), indicating a moderate quality overall (see Supplementary materials for summary tables). The reporting domain (*M *= 5.41, *SD *= 0.94) and internal validity (*M *= 2.91, *SD *= 0.74) were strengths. However, many studies did not report the validity and reliability of measures used (*k *= 35) and few provided the response rate from recruitment (*k *= 18). External validity was poorer (*M *= 1.45, *SD *= 0.84), with only 20 studies randomly sampling or consecutively recruiting participants. Very few studies (*k *= 6) used power analyses. The confidence in each analysis was also assessed using GRADE criteria (see Supplementary materials for summary tables). Out of the 20 analyses, the confidence in 16 outcomes was downgraded for at least one domain. Imprecision was the most common reason for downgrading (*n* = 10), due to the wide 95% CIs undermining confidence in the direction or size of the effect found. Inconsistency was another common reason evidence was downgraded (*n* = 7), as there was often high heterogeneity in analyses. Lastly, five analyses were downgraded due to concerns about risk of bias as a high proportion of contributing studies scored poorly on the QIS.

### Sensitivity analyses

A post hoc sensitivity analysis was conducted and removed nine studies published before or during 2000, before the introduction of biologic medications. The pattern of results was largely unchanged, with the only difference being that JIA groups no longer had fewer externalizing problems compared to other illnesses (*g *= −0.06, 95% CI −0.46 to 0.34, *p *= .774) Another post hoc sensitivity analysis was performed to exclude studies published before or during 2003, when LeBovidge’s (2003) meta-analysis was published. This resulted in 12 studies being removed from the original analyses. There were minimal changes to results when excluding these studies, however, as with the previous sensitivity analysis, there was no longer a difference in the externalizing problems between youth with JIA and other illnesses (*g *= 0.01, 95% CI −0.58 to 0.78, *p* = .779). See Supplementary materials for full results of sensitivity analyses.

## Discussion

The aim of this meta-analysis was to determine the impact of JIA on psychosocial outcomes and HRQoL. We found internalizing problems and psychiatric diagnoses were greater among youth with JIA than among youth without JIA, but comparable to youth with other chronic illnesses. Conversely, youth with JIA had comparable levels of anxiety and depressive symptoms and externalizing problems compared to youth without JIA, and lower levels than in other illnesses, indicating resilience. Unsurprisingly, youth with JIA had much greater levels of pain and functional disability compared to those without JIA. Consistent with this, a moderate to large deficit was observed across all HRQoL domains, compared to youth without JIA. Although youth with JIA did not have greater pain or worse functional ability than youth with other illnesses, those with JIA nevertheless experienced poorer physical and psychosocial HRQoL.

These findings are consistent with a previous meta-analysis conducted more than two decades ago (LeBovidge et al., 2003), which likewise found that youth with JIA had more internalizing problems than healthy peers but a similar level of externalizing problems. That meta-analysis did not examine anxiety and depressive symptoms in youth with JIA, but rather only investigated broad measures of internalizing problems. Interestingly, our meta-analysis found that youth with JIA did not report more anxiety and depressive symptoms than youth without JIA and reported fewer symptoms than those with other illnesses.

This finding is in contrast to the results reported by Fair and colleagues (2019), who concluded that youth with JIA had more depression and anxiety. However, their review was based on rates of diagnosed anxiety and depressive disorders, while the studies included in our depression and anxiety outcomes used questionnaire data that assessed anxiety and depressive symptoms. As such, their results are consistent with our findings of higher rates of psychiatric diagnoses among those with JIA compared to healthy controls. This suggests that JIA does not contribute to increased symptoms of depression and anxiety across all youth with JIA; however, more youth with JIA report a sufficiently severe level of mental health symptoms to meet diagnostic criteria for a psychiatric disorder. Specifically, it is worth considering that the elevated rates of psychiatric diagnoses may also reflect other psychiatric disorders, such as eating disorders or post-traumatic stress disorders, which would not be captured by anxiety and depression specific questionnaires. Additionally, the questionnaires frequently used to assess anxiety and depressive symptoms have been critiqued in the past for lacking diagnostic sensitivity (e.g., Hodges, 1990; Roseman et al., 2016), which could also be contributing to this discrepancy in findings. Considering these findings together, a more comprehensive mental health screening process should be implemented as standard in clinical practice so that youth with JIA in distress can be identified and referred to treatment.

It is possible that these lower levels of anxiety, depressive, and externalizing symptoms in youth with JIA relative to other chronic illnesses, reflect better adaptation to the condition. In support of this, LeBovidge et al (2005) found that 80% of the 75 youth with JIA in their study reported positive aspects of their experience with arthritis, such as greater determination and resilience and developing closer relationships with others. These more positive attitudes toward their illness also predicted lower anxiety and depressive symptoms. As such, this more optimistic focus could be contributing to better psychological adjustment relative to other chronic illness.

Whereas, the most represented comparator illness conditions, diabetes, SCD, and CFS each carry unique challenges which could contribute to poorer psychological adjustment. For example, youth with type 1 diabetes report stress around daily blood glucose management demands (Davidson et al., 2004) and anxiety about the risk of hypoglycemia (Driscoll et al., 2016). SCD features unpredictable and intense pain episodes, and frequent hospitalization, both of which have been linked to increased depression (Grant et al., 2000). Lastly, youth with CFS were found to have more illness-related worries and poorer coping strategies compared to those with JIA (Garralda & Rangel, 2004) and frequently report experiences of invalidation and stigmatization (Parslow et al., 2017). Together, these challenges, alongside the relative resilience of youth with JIA, may account for the better psychological adjustment found in youth with JIA.

Given evidence of resilience of youth with JIA in terms of anxiety and depressive symptoms in the face of pain and disability, why might youth with JIA nevertheless have such poor HRQoL? In 2011, there were just 645 pediatric rheumatologists worldwide, a number that could only meet 11.5% of the estimated demand (Henrickson, 2011). These shortages extend to countries with comparatively accessible healthcare systems such as Australia and New Zealand (Cox et al., 2017) as well as the United Kingdom (McErlane et al., 2016). Consequently, youth with JIA often wait a long time to receive a diagnosis. A global review found a median wait time of 5.78 months from symptom onset to first pediatric rheumatology appointment (Chausset et al., 2020). Further, youth diagnosed with JIA had seen a median of three health professionals before being referred appropriately (Chausset et al., 2020). This delay is in stark contrast to guidelines which recommend they are examined by a pediatric rheumatologist within ten weeks of symptom onset (Davies et al., 2010) and is detrimental in the long term as treatments are most efficacious in these early stages (Albers et al., 2009; Sherry et al., 1999). Research confirms that delayed diagnosis is associated with worse functional abilities and poorer HRQoL among youth six months later (Oen et al., 2009). Disease remission is also lower among those who waited 12 months for a referral to specialized treatment (Fantini et al., 2003). Similarly, although multidisciplinary treatment is recommended (Davies et al., 2010), it is rarely implemented (Cox et al., 2017; Lee et al., 2022; Singh-Grewal, 2017). These challenges in accessing healthcare may contribute to the poorer HRQoL in youth with JIA and importantly, may be modifiable. For instance, the broader adoption of a multidisciplinary model of care could enhance the support provided to these young people and prevent longer-term poorer HRQoL.

Several moderators were identified in this study. We found that older JIA samples had fewer psychiatric diagnoses and better HRQoL, when compared to other illness groups. Similarly, we found that greater disease duration was associated with less depressive symptoms compared to youth without JIA. Together, these may indicate that youth with JIA experience an adjustment in their psychosocial outcomes over time. However, we also found evidence to suggest that a longer disease duration may elicit greater internalizing problems relative to healthy peers. This contradictory finding could be due to measurement issues. Specifically, others have detailed how the CBCL’s internalizing scale includes items which assess somatic complaints, which could result in inflated reports of internalizing problems among youth with chronic illnesses (LeBovidge et al., 2003; Perrin et al., 1991). Taking this into account, it could be that over time more somatic symptoms are experienced by youth with JIA, but fewer psychological symptoms.

While we attended carefully to the methodology of this study, this review was not without limitations. Firstly, although we completed an extensive search of multiple databases, we did not search the gray literature, which may have resulted in the exclusion of some eligible unpublished studies. We also limited the inclusion criteria to studies published in English. As a result, this may limit the generalizability of our results to non-English speaking populations. Moreover, although we contacted authors to retrieve missing data, not all attempts were successful, and it is possible as a result, some relevant data were unable to be retrieved and included in our analyses.

Another potential limitation is the lack of diversity in studies, which may limit the generalizability of these results. All studies that reported on the race and ethnic background of participants had a majority White/Caucasian sample. However, it is also worth noting that most studies (*n* = 40) did not report any data on the racial or ethnic background of participants. Similarly, less than half of all studies (*n* = 26) provided any information on the socioeconomic status (SES) of their participants. When they did, participants predominantly came from middle or higher SES backgrounds. This may also limit the generalizability of results to youth from lower SES backgrounds. However, the lack of reporting also makes it difficult to assess the generalizability of results and demonstrates the importance of future research prioritizing the collecting and reporting this information. Compounding this, most studies were conducted in either European countries (*k *= 25) or the United States (*k *= 17). Considering that access to treatment for JIA is poorer in African and Asian countries (Henrickson, 2011), these findings may under-estimate the psychosocial impact of JIA on youth globally.

Moreover, many of the studies in this review used small samples, with 31 studies including <50 youth with JIA. These small sample sizes may have contributed to the high number of analyses that were downgraded due to lack of precision, as CIs were often wide limiting certainty in the size of the effect. Heterogeneity was also frequently high, resulting in several outcomes being downgraded for inconsistency. Some of this heterogeneity was explained by the moderating effects of age and disease duration on outcomes. However, this heterogeneity was likely also contributed to my other variables that we lacked sufficient data to explore, such as differences in the type of illness control groups. Lastly, studies had poorer external validity and commonly relied on convenience samples, which may not be representative. Aligned with this, the confidence in five analyses was downgraded due to the risk of bias.

Additionally, as raised previously, the CBCL, which was commonly used to measure internalizing problems, may have inflated the report of internalizing problems among youth with JIA. However, youth with JIA were still found to be more likely to have psychiatric disorders than their healthy peers. As such, although the evidence in favor of differences in internalizing symptoms is less compelling, at the diagnosable level, psychiatric disorders more commonly affect youth with JIA relative to their healthy peers.

These limitations notwithstanding, this review is the first to meta-analysis HRQoL outcomes and provides an important update on the impact of JIA on psychological outcomes, including a sensitivity analysis since the introduction of biologic treatments. Evidence suggests that youth with JIA have more psychiatric disorders and may have more internalizing problems than healthy peers but show psychological resilience, having symptoms of anxiety and depression that are overall comparable to their healthy peers, and at a lower severity than other illness groups. Importantly, despite these patterns indicating resilience, youth with JIA face considerably poorer HRQoL outcomes, with large deficits in physical and psychosocial quality of life, even relative to other illnesses. These outcomes may partly be the product of the issues in accessing pediatric rheumatology services early in the disease trajectory. The under-resourcing of youth with JIA in the healthcare system parallels the absence of research about this population. This is exemplified by the fact that just 62.5% of studies included in this review referred to youth with arthritis in their titles. Many studies instead featured youth with JIA as a control group to investigate outcomes for youth with other chronic conditions. There is a clear need for more research directed toward better understanding the unmet needs of youth with JIA. Current evidence suggests that youth with JIA do adjust psychologically over time, despite high levels of pain and disability. Research is needed to identify how to further facilitate adjustment in the early stages of illness to best support these young people to achieve a better quality of life. Promisingly, Stinson and colleagues’ (2020) self-management program was found to improve HRQoL in adolescents with JIA. This suggests that targeted psychosocial interventions may help to alleviate the HRQoL deficit in youth with JIA and that the development and evaluation of these interventions should be a priority for future research. Lastly, a greater emphasis should be placed on mental health screening and implementing a multidisciplinary model of care to more holistically support youth with JIA.

## Supplementary Material

jsaf067_Supplementary_Data

## Data Availability

Data will be available upon reasonable request to the corresponding author.
