# Supplementary material for: The impact of juvenile idiopathic arthritis on psychosocial outcomes: a systematic review and meta-analysis
Source: J Pediatr Psychol. 2025 Aug 5;50(11):1033–49. doi: 10.1093/jpepsy/jsaf067 (PMC12633853; doi:10.1093/jpepsy/jsaf067)
Supplement: jsaf067_Supplementary_Data [file jsaf067_supplementary_data.docx]

**Supplementary Material**

**Table 1.** Database Search Strategy Examples

**Table 2**. Quality Index Scores

**Table 3**. Risk of Bias Summary Table

**Table 4**. Summary of GRADE Evaluation of Analyses Comparing JIA to Healthy Controls

**Table 5**. Summary of GRADE Evaluation of Analyses Comparing JIA to Illness Controls

**Table 6**. Healthy Controls Sensitivity Analyses (Filtering out before 2000)

**Table 7**. Healthy Controls Sensitivity Analyses (Filtering out before 2000)

**Table 8**. Illness Controls Sensitivity Analyses (Filtering out before 2000)

**Table 9**. Illness Controls Sensitivity Analyses (Filtering out before 2003)

**Figure 1.** Forest Plot of Internalising Problems compared to Healthy Groups

**Figure 2.** Forest Plot of Psychiatric Diagnoses compared to Healthy Groups

**Figure 3.** Forest Plot of Anxiety compared to Healthy Groups

**Figure 4.** Forest Plot of Depression compared to Healthy Groups

**Figure 5.** Forest Plot of Externalising compared to Healthy Groups

**Figure 6.** Forest Plot of Total HRQoL compared to Healthy Groups

**Figure 7.** Forest Plot of Physical HRQoL compared to Healthy Groups

**Figure 8.** Forest Plot of Psychosocial HRQoL compared to Healthy Groups

**Figure 9.** Forest Plot of Pain compared to Healthy Groups

**Figure 10.** Forest Plot of Functional Disability compared to Healthy Groups

**Figure 11.** Forest Plot of Internalising compared to Illness Groups

**Figure 12.** Forest Plot of Psychiatric Diagnoses compared to Illness Groups

**Figure 13.** Forest Plot of Anxiety compared to Illness Groups

**Figure 14.** Forest Plot of Depression compared to Illness Groups

**Figure 15.** Forest Plot of Externalising compared to Illness Groups

**Figure 16.** Forest Plot of Total HRQoL compared to Illness Groups

**Figure 17.** Forest Plot of Physical HRQoL compared to Illness Groups

**Figure 18.** Forest Plot of Psychosocial HRQoL compared to Illness Groups

**Figure 19.** Forest Plot of Pain compared to Illness Groups

**Figure 20.** Forest Plot of Functional Disability compared to Illness Groups

**Table 1**. Database Search Strategy Examples

| Scopus | |
| --- | --- |
| 1 | TITLE-ABS((“juvenile arthritis” OR “juvenile idiopathic arthritis” OR “juvenile chronic arthritis” OR “juvenile rheumatoid arthritis” OR “juvenile rheumatic disease”)) |
| 2 | TITLE-ABS((psych* OR psychosocial OR depress* OR anxi* OR pain OR “quality of life”*)) |
| 3 | #1 AND #2 |
| 4 | Limit to English language |
| PsycINFO | |
| 1 | juvenile arthritis.mp. |
| 2 | juvenile idiopathic arthritis.mp. |
| 3 | juvenile chronic arthritis.mp. |
| 4 | juvenile rheumatoid arthritis.mp. |
| 5 | juvenile rheumatic disease.mp. |
| 6 | psych*.mp. |
| 7 | psychosocial.mp. or exp psychosocial outcomes/ or exp psychosocial factors/ |
| 8 | exp Anxiety/ or anxi*.mp. |
| 9 | Exp Major Depression/ or depress*.mp. |
| 10 | Exp pain/ or pain.mp. or exp chronic pain/ |
| 11 | Quality of life.mp or exp “Quality of Life”/ |
| 12 | 1 or 2 or 3 or 4 or 5 |
| 13 | 6 or 7 or 8 or 9 or 10 or 11 |
| 14 | 12 and 13 |
| 15 | Limit 14 to (peer reviewed journal and english language) |
| PubMED | |
| 1 | (Juvenile arthritis[Title/Abstract] OR juvenile idiopathic arthritis[Title/Abstract] OR juvenile chronic arthritis[Title/Abstract] OR juvenile rheumatoid arthritis[Title/Abstract] OR juvenile rheumatic diseaseTitle/Abstract] |
| 2 | (psych*[Title/Abstract] OR psychosocial[Title/Abstract] OR anxi*[Title/Abstract OR depress*[Title/Abstract] OR pain[Title/Abstract] OR quality of life[Title/Abstract] |
| 3 | #1 AND #2 |
| 4 | Only English |

**Table 2**. Quality Index Scores

| Study | Reporting | External Validity | Internal Validity | Power | Total QIS |
| --- | --- | --- | --- | --- | --- |
| Aasland 1997 | 3 | 1 | 3 | 0 | 7 |
| Aasland 1999 | 5 | 1 | 4 | 0 | 10 |
| Abdelaleem 2021 | 5 | 0 | 2 | 0 | 7 |
| Bomba 2013 | 4 | 1 | 3 | 0 | 8 |
| Brace 2000 | 7 | 1 | 3 | 0 | 11 |
| Butbul Aviel 2011 | 7 | 2 | 4 | 0 | 13 |
| Butler 2018 | 7 | 2 | 4 | 0 | 13 |
| Castaneda 2013 | 7 | 3 | 3 | 0 | 13 |
| Conte 2003 | 5 | 2 | 2 | 0 | 9 |
| Delcoigne 2023 | 5 | 3 | 3 | 0 | 11 |
| Feldmann 2005 | 4 | 1 | 2 | 0 | 7 |
| Filocamo 2010 | 6 | 2 | 4 | 0 | 12 |
| Fischer 2019 | 6 | 1 | 3 | 0 | 10 |
| Fontecha 2011 | 5 | 0 | 4 | 1 | 10 |
| Frank 1998 | 5 | 1 | 4 | 0 | 10 |
| Fuchs 2013 | 6 | 1 | 3 | 0 | 10 |
| Gartstein 1999 | 6 | 2 | 2 | 1 | 11 |
| Gray 2001 | 5 | 1 | 2 | 0 | 8 |
| Graziano 2016 | 4 | 2 | 4 | 0 | 10 |
| Guerriero 2022 | 6 | 1 | 3 | 0 | 10 |
| Haines 2019 | 5 | 1 | 4 | 1 | 11 |
| Haverman 2012 | 6 | 2 | 4 | 0 | 12 |
| Huygen 2000 | 5 | 1 | 3 | 0 | 9 |
| Janicke 2008 | 5 | 3 | 2 | 0 | 10 |
| Kayan Ocakoglu 2018 | 4 | 1 | 2 | 0 | 7 |
| Kuburovic 2014 | 6 | 1 | 4 | 0 | 11 |
| Kwon 2015 | 5 | 1 | 2 | 0 | 8 |
| Kyllonen 2021 | 4 | 3 | 2 | 0 | 9 |
| Listing 2018 | 5 | 2 | 3 | 0 | 10 |
| Long 2008 | 6 | 1 | 3 | 0 | 10 |
| Lundberg 2017 | 6 | 2 | 3 | 0 | 11 |
| McDonald 2022 | 6 | 1 | 3 | 0 | 10 |
| Milatz 2024 | 5 | 3 | 2 | 0 | 10 |
| Mullick 2005 | 5 | 2 | 3 | 0 | 10 |
| Noll 2000 | 7 | 2 | 3 | 1 | 13 |
| Ojmyr-Joelsson 2006 | 4 | 0 | 2 | 0 | 6 |
| Oliveira 2007 | 6 | 0 | 2 | 0 | 8 |
| Oymak 2015 | 4 | 1 | 2 | 0 | 7 |
| Pedersen 2024 | 5 | 3 | 3 | 0 | 11 |
| Peterson 2004 | 6 | 1 | 4 | 0 | 11 |
| Polat 2023 | 6 | 1 | 3 | 0 | 10 |
| Rangel 2003 | 6 | 1 | 3 | 0 | 10 |
| Reid 1997 | 5 | 1 | 2 | 1 | 9 |
| Ringold 2009 | 7 | 2 | 3 | 0 | 12 |
| Scott 2018 | 5 | 1 | 2 | 0 | 8 |
| Selvaag 2005 | 7 | 3 | 2 | 0 | 12 |
| Shaaban 2006 | 5 | 1 | 2 | 0 | 8 |
| Sikorová 2016 | 5 | 1 | 2 | 0 | 8 |
| Tarakci 2011 | 6 | 1 | 3 | 1 | 11 |
| Tsipoura 2018 | 6 | 1 | 3 | 0 | 10 |
| Wagner 2007 | 5 | 1 | 3 | 0 | 9 |
| Wallander 1988 | 5 | 1 | 3 | 0 | 9 |
| Ward 2017 | 6 | 2 | 3 | 0 | 11 |
| Ward 2014 | 4 | 0 | 4 | 0 | 8 |
| Weitzman 2023 | 6 | 3 | 3 | 0 | 12 |
| Zebracki 2004 | 6 | 2 | 4 | 0 | 12 |

**
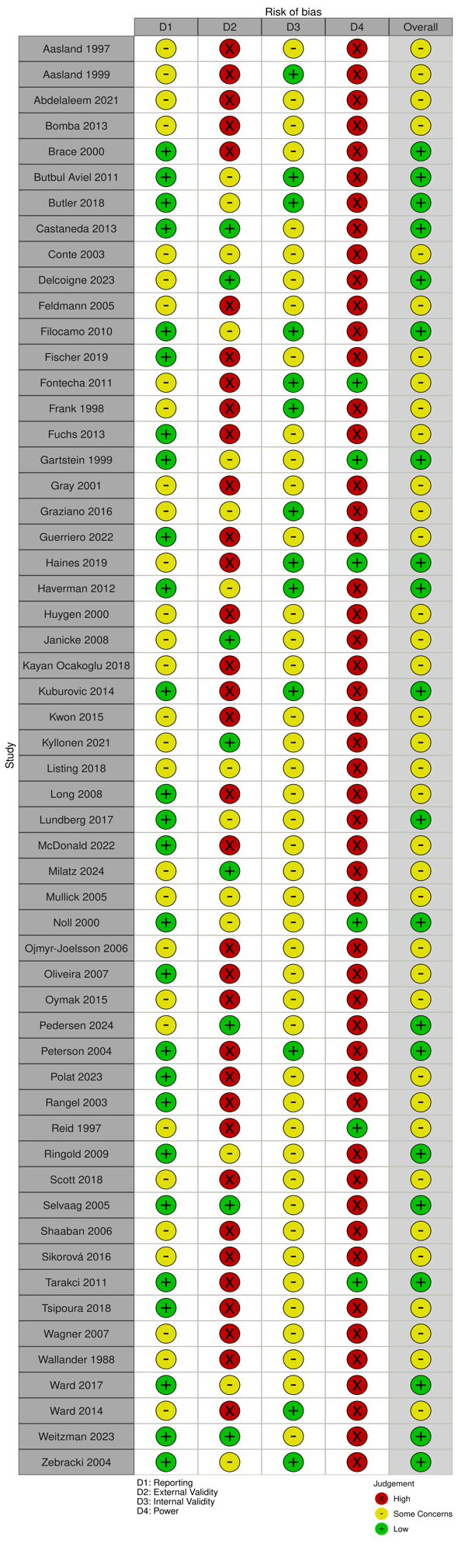
Table 3**. Risk of Bias Summary Table

**Table 4**. Summary of GRADE Evaluation of Analyses Comparing JIA to Healthy Controls

| Outcome | Risk of Bias | Inconsistency | Imprecision | Publication bias | Quality |
| --- | --- | --- | --- | --- | --- |
| Internalising | Low | Not serious | Not serious | Not serious | ⨁⨁⨁⨁ |
| Psychiatric Diagnoses | Low | Serious | Not serious | Not serious | ⨁⨁⨁⊝^a^ |
| Anxiety | Low | Not serious | Serious | Not serious | ⨁⨁⨁⊝^b^ |
| Depression | Low | Not serious | Serious | Not serious | ⨁⨁⨁⊝^b^ |
| Externalising | Some Concerns | Not serious | Serious | Not serious | ⨁⨁⊝^b^⊝^c^ |
| Total HRQoL | Low | Serious | Not serious | Not serious | ⨁⨁⨁⊝^a^ |
| Physical HRQoL | Low | Serious | Not serious | Not serious | ⨁⨁⨁⊝^a^ |
| Psychosocial HRQoL | Low | Serious | Not serious | Not serious | ⨁⨁⨁⊝^a^ |
| Pain | Low | Not serious | Not serious | Not serious | ⨁⨁⨁⨁ |
| Functional Abilities | Some concerns | Not serious | Not serious | Not serious | ⨁⨁⨁⊝^c^ |

*Note*. ^a^ = Downgraded for inconsistency (>50% of the studies were outside of the 95% CI and I^2^ >75%); ^b^ = Downgraded for imprecision (the interpretation of results differed if upper or lower 95% CI reflected the true effect or the total participants were lower than the calculated OIS); ^c^ = Downgraded for risk of bias (>75% of studies were evaluated as ‘Some Concerns’ or ‘High Risk’).

**Table 5**. Summary of GRADE Evaluation of Analyses Comparing JIA to Illness Controls

| Outcome | Risk of Bias | Inconsistency | Imprecision | Publication bias | Quality |
| --- | --- | --- | --- | --- | --- |
| Internalising | Some concerns | Not serious | Serious | Not serious | ⨁⨁⊝^a^⊝^b^ |
| Psychiatric Diagnoses | Some concerns | Not serious | Serious | Not serious | ⨁⨁⊝^a^⊝^b^ |
| Anxiety | Low | Not serious | Not serious | Not serious | ⨁⨁⨁⨁ |
| Depression | Low | Not serious | Not serious | Not serious | ⨁⨁⨁⨁ |
| Externalising | Some concerns | Not serious | Not serious | Not serious | ⨁⨁⨁⊝^a^ |
| Total HRQoL | Low | Not serious | Serious | Not serious | ⨁⨁⨁⊝^b^ |
| Physical HRQoL | Low | Serious | Serious | Not serious | ⨁⨁⊝^b^⊝^c^ |
| Psychosocial HRQoL | Low | Not serious | Serious | Not serious | ⨁⨁⨁⊝^b^ |
| Pain | Low | Serious | Serious | Not serious | ⨁⨁⊝^b^⊝^c^ |
| Functional Abilities | Low | Serious | Serious | Not serious | ⨁⨁⊝^b^⊝^c^ |

*Note*. ^a^ = Downgraded for risk of bias (>75% of studies were evaluated as ‘Some Concerns’ or ‘High Risk’); ^b^ = Downgraded for imprecision (the interpretation of results differed if upper or lower 95% CI reflected the true effect OR total participants were lower than calculated OIS); ^c^ = Downgraded for inconsistency (>50% of the studies were outside of the 95% CI and I^2^ >75%).

**Table 6**. Healthy Controls Sensitivity Analyses Filtering out Studies Published During or Before 2000

| Outcome | No. of Studies | No. of Effects | Effect size (95% CI) | *P* | *Q* | *I^2^* |
| --- | --- | --- | --- | --- | --- | --- |
| Internalising | 7 | 8 | 0.31 (0.04, 0.58) | 0.023 | 10.19 | 31.28 |
| Anxiety | 5 | 5 | 0.28 (-0.16, 0.72) | 0.216 | 11.55 | 65.37 |
| Depression | 5 | 5 | 0.36 (-0.02, 0.74) | 0.060 | 7.65 | 47.73 |
| Externalising | 7 | 8 | 0.21 (-0.01, 0.44) | 0.060 | 7.67 | 8.74 |
| Pain | 7 | 7 | 1.26 (1.08, 1.45) | <.001 | 26.57 | 77.41 |
| Functional Ability | 6 | 6 | -1.22 (-1.50, -0.95) | <0.001 | 13.67 | 63.42 |

*Note*. Sensitivity analyses were not performed for all HRQoL outcomes and psychiatric diagnoses as no study published on or before 2000 were included in original analyses.

**Table 7**. Healthy Controls Sensitivity Analyses Filtering out Studies Published During or Before 2003

| Outcome | No. of Studies | No. of Effects | Effect size (95% CI) | *P* | *Q* | *I^2^* |
| --- | --- | --- | --- | --- | --- | --- |
| Internalising | 6 | 7 | 0.35 (0.05, 0.64) | 0.022 | 9.83 | 38.96 |
| Anxiety | 4 | 4 | 0.38 (-0.09, 0.85) | 0.113 | 9.23 | 67.49 |
| Depression | 4 | 4 | 0.39 (-0.07, 0.85) | 0.097 | 7.60 | 60.53 |
| Externalising | 6 | 7 | 0.23 (-0.03, 0.48) | 0.082 | 7.63 | 21.39 |
| Pain | 7 | 7 | 1.26 (1.08, 1.45) | <.001 | 26.57 | 77.41 |
| Functional Ability | 6 | 6 | -1.22 (-1.50, -0.95) | <0.001 | 13.67 | 63.42 |

*Note*. Sensitivity analyses were not performed for all HRQoL outcomes and psychiatric diagnoses as no study published on or before 2003 were included in original analyses.

**Table 8**. Illness Controls Sensitivity Analyses Filtering out Studies Published During or Before 2000

| Outcome | No. of Studies | No. of Effects | Effect size (95% CI) | *P* | *Q* | *I^2^* |
| --- | --- | --- | --- | --- | --- | --- |
| Internalising | 7 | 9 | -0.35 (-1.41, 0.70) | 0.513 | 215.08 | 96.28 |
| Psychiatric Diagnoses | 4 | 9 | -0.08 (-0.22, 0.06) | 0.256 | 13.75 | 41.81 |
| Anxiety | 6 | 8 | -0.27 (-0.43, -0.11) | 0.001 | 5.39 | 0 |
| Depression | 8 | 10 | -0.45 (-0.64, -0.26) | <.001 | 12.48 | 27.87 |
| Externalising | 6 | 7 | -0.06 (-0.46, 0.34) | 0.774 | 11.07 | 45.80 |
| Pain | 4 | 7 | -0.31 (-0.98, 0.36) | 0.367 | 58.35 | 89.72 |
| Functional Ability | 3 | 5 | 0.09 (-0.57, 0.39) | 0.719 | 26.96 | 85.16 |

*Note*. Sensitivity analyses were not performed for all HRQoL outcomes as no study published on or before 2000 were included in original analyses.

**Table 9**. Illness Controls Sensitivity Analyses Filtering out Studies Published During or Before 2003

| Outcome | No. of Studies | No. of Effects | Effect size (95% CI) | *P* | *Q* | *I^2^* |
| --- | --- | --- | --- | --- | --- | --- |
| Internalising | 4 | 6 | 0.008 (-1.41, 1.43) | 0.991 | 188.38 | 97.35 |
| Psychiatric Diagnoses | 3 | 8 | -0.05 (-0.15, 0.05) | 0.345 | 7.35 | 4.69 |
| Anxiety | 5 | 7 | -0.24 (-0.40, -0.07) | 0.005 | 1.26 | 0 |
| Depression | 7 | 9 | -0.41 (-0.59, -0.24) | <.001 | 9.56 | 16.29 |
| Externalising | 3 | 4 | 0.01 (-0.58, 0.78) | 0.779 | 7.60 | 60.55 |
| Pain | 4 | 7 | -0.31 (-0.98, 0.36) | 0.367 | 58.35 | 89.72 |
| Functional Ability | 3 | 5 | 0.09 (-0.57, 0.39) | 0.719 | 26.96 | 85.16 |

*Note*. Sensitivity analyses were not performed for all HRQoL outcomes as no study published on or before 2003 were included in original analyses.

**Figure 1.** Forest Plot of Internalising Problems compared to Healthy Groups

**Figure 2.** Forest Plot of Psychiatric Diagnoses compared to Healthy Groups

**Figure 3.** Forest Plot of Anxiety compared to Healthy Groups

**Figure 4.** Forest Plot of Depression compared to Healthy Groups

**Figure 5.** Forest Plot of Externalising compared to Healthy Groups

**Figure 6.** Forest Plot of Total HRQoL compared to Healthy Groups
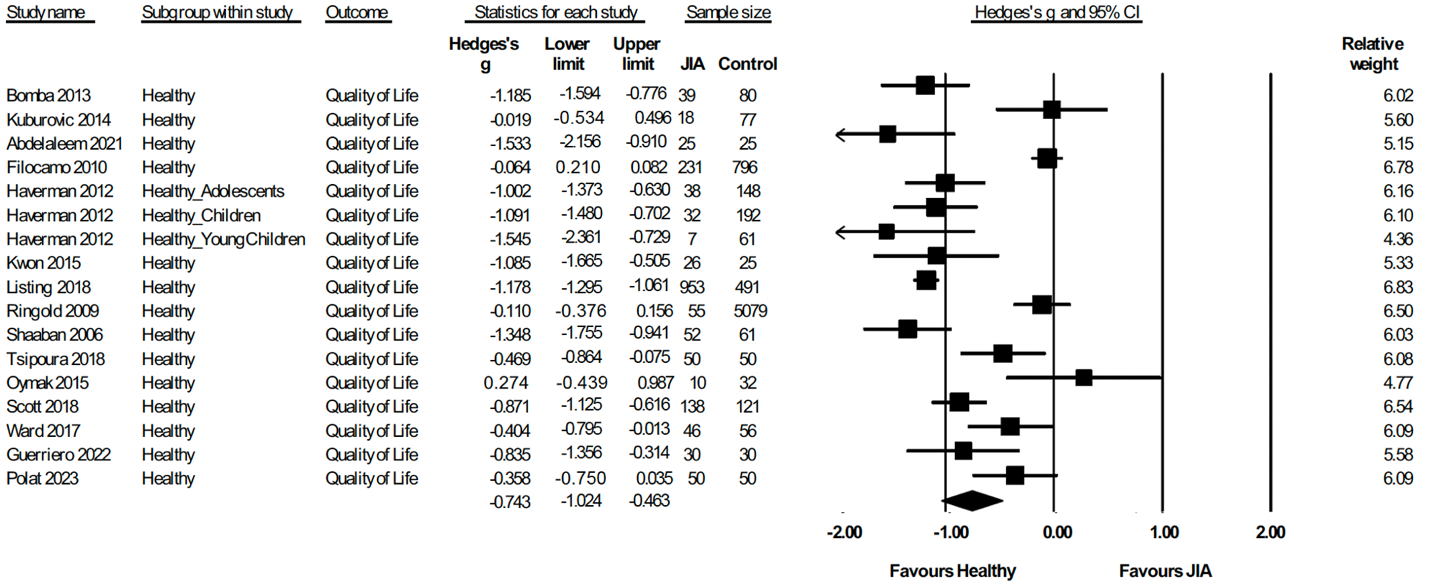


**
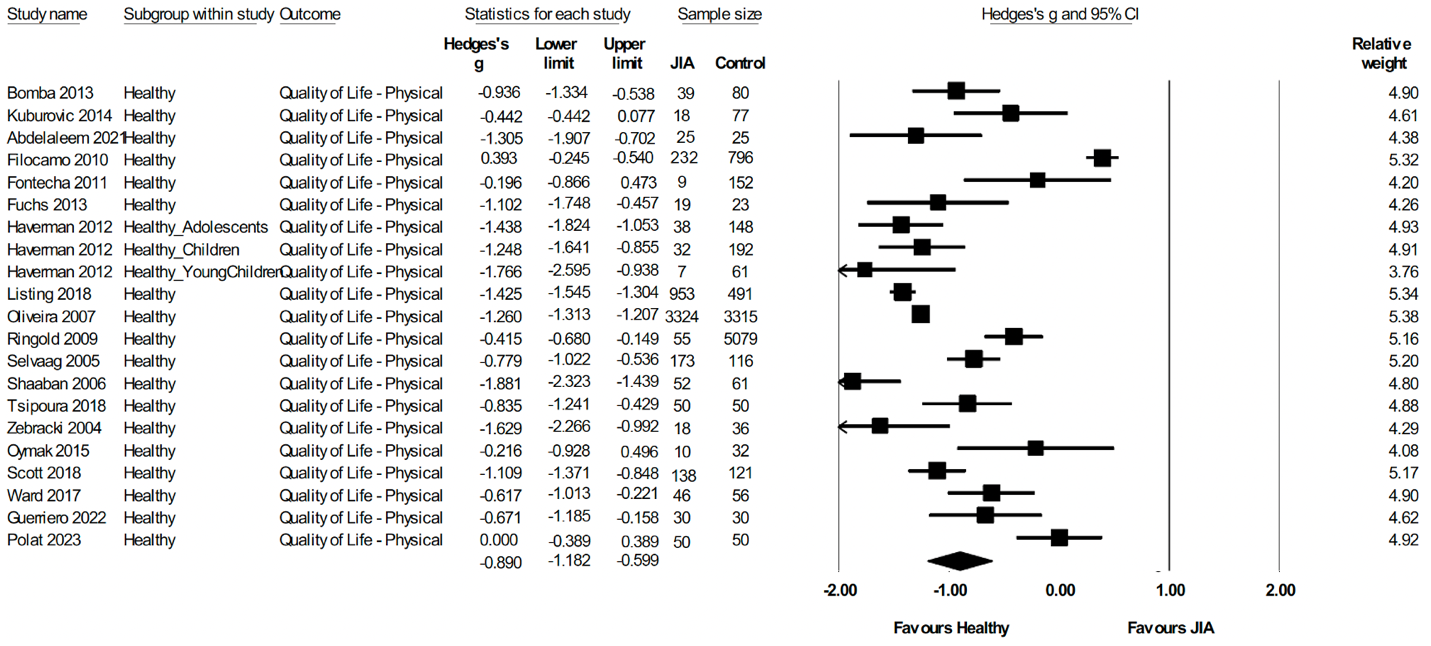
Figure 7.** Forest Plot of Physical HRQoL compared to Healthy Groups

**
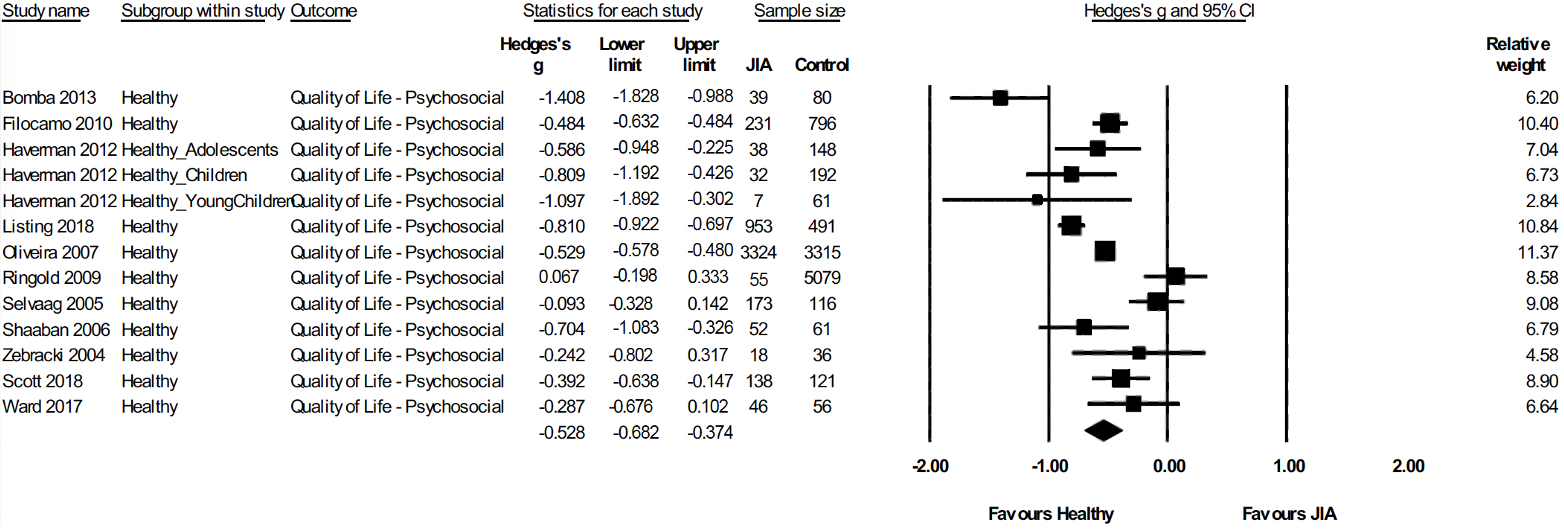
Figure 8.** Forest Plot of Psychosocial HRQoL compared to Healthy Groups

**Figure 9.** Forest Plot of Pain compared to Healthy Groups

**Figure 10.** Forest Plot of Functional Disability compared to Healthy Groups

**Figure 11.** Forest Plot of Internalising compared to Illness Groups

*Note*. SCD = Sickle Cell Disease.

**Figure 12.** Forest Plot of Psychiatric Diagnoses compared to Illness Groups

*Note*. Metabolic = Metabolic Syndrome; SCD = Sickle Cell Disease.

**Figure 13.** Forest Plot of Anxiety compared to Illness Groups

*Note.* CFS = Chronic Fatigue Syndrome; SCD = Sickle Cell Disease.

**Figure 14.** Forest Plot of Depression compared to Illness Groups

*Note.* CFS = Chronic Fatigue Syndrome; SCD = Sickle Cell Disease.

**Figure 15.** Forest Plot of Externalising compared to Illness Groups

*Note.* SCD = Sickle Cell Disease.

**Figure 16.** Forest Plot of Total HRQoL compared to Illness Groups


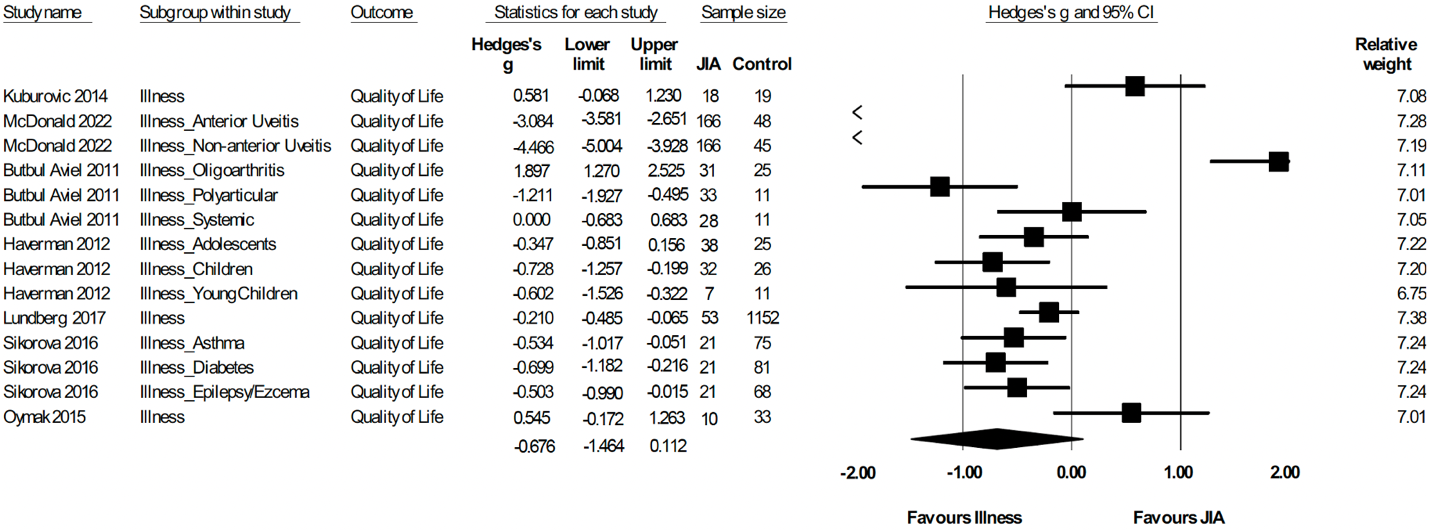


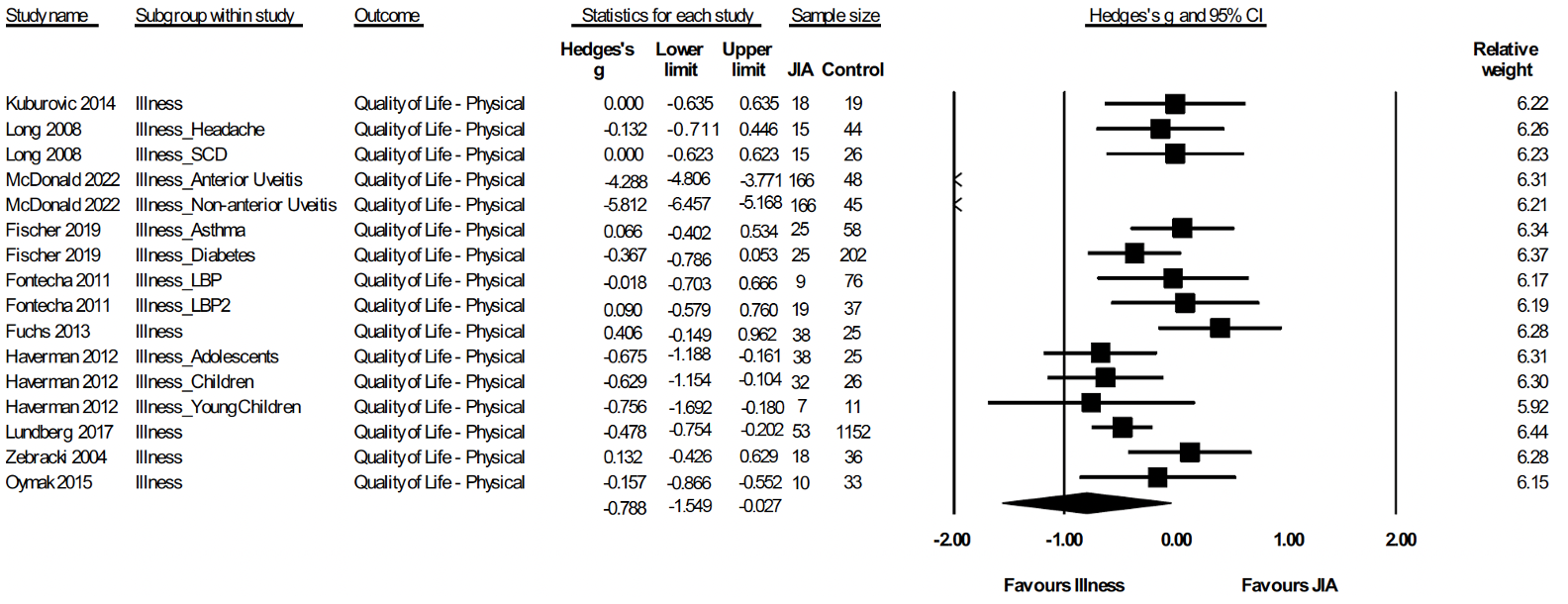
**Figure 17.** Forest Plot of Physical HRQoL compared to Illness Groups

*Note.* SCD = Sickle Cell Disease; LBP = Low Back Pain Group 1; LBP2= Low Back Pain Group 2.


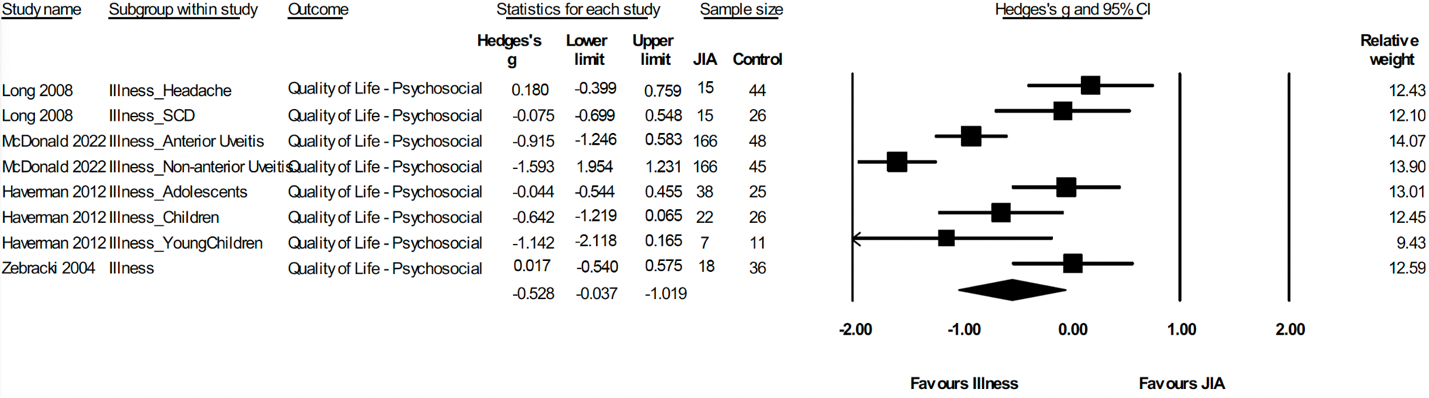
**Figure 18.** Forest Plot of Psychosocial HRQoL compared to Illness Groups

*Note.* SCD = Sickle Cell Disease.

**Figure 19.** Forest Plot of Pain compared to Illness Groups

*Note.* SCD = Sickle Cell Disease.

**Figure 20.** Forest Plot of Functional Disability compared to Illness Groups

*Note.* SCD = Sickle Cell Disease.
